# Biomechanics in DALK: Big bubble vs manual lamellar dissection

**DOI:** 10.5935/0004-2749.20200022

**Published:** 2020

**Authors:** Mehmet Orcun Akdemir, Banu Torun Acar, Suphi Acar

**Affiliations:** 1 Department of Ophthalmology, School of Medicine, Bulent Ecevit University, Zonguldak, Turkey; 2 Haydarpasa Numune Research and Education Hospital, Istanbul, Turkey

**Keywords:** Cornea/physiopathology, Corneal transplantation/ methods, Córnea/fisiopatologia, Transplante de córnea/ métodos

## Abstract

**Purpose:**

The present study’s aim was to compare the biomechanical properties of corneal tissue in patients who underwent deep anterior lamellar keratoplasty (DALK) surgery, with successful big bubble formation and manual lamellar dissection, during failed big bubble formation.

**Methods:**

This retrospective comparative study included 60 eyes from 60 keratoconus patients who previously underwent DALK surgery. These patients were categorized as big bubble (+) or big bubble (-) based on the success or failure of big bubble formation during the surgery. The big bubble (+) group included 42 eyes, while the big bubble (-) group had 18 eyes. Moreover, the patients were regrouped as 0.25 mm and 0.50 mm to evaluate the effects of the disparity between donor and trephine punches on the biomechanical properties of the cornea. These biomechanical properties, characterized by corneal hysteresis and the corneal resistance factor, were measured using the Ocular Response Analyzer 12 months after the surgery.

**Results:**

There was no statistically significant difference between the big bubble (+) and big bubble (-) groups in the biomechanical properties of the cornea (corneal hysteresis: 10.06, 10.25; p=0.716/corneal resistance factor: 10.15, 10.07; p=0.805, respectively). In addition, pachymetry results were not statistically different between the two groups. Multivariate regression analysis revealed that corneal hysteresis and corneal resistance factor were positively associated with central corneal thickness (p<0.001/r^2^=0.506; p<0.001/ r^2^=0.561, respectively). However, the study did not demonstrate a relationship between any of the punch sizes and corneal hysteresis or between the punch sizes (p=0.673) and the corneal resistance factor (p=0.643).

**Conclusions:**

The corneal hysteresis and corneal resistance factor values were similar in big bubble and manual lamellar dissection after DALK. Thus, manual lamellar dissection was not a disadvantage considering the cornea’s biomechanical properties.

## Abstract (pt)

**Objetivo:**

O objetivo do estudo foi comparar parâmetros biomecânicos corneanos de pacientes com cirurgia de ceratoplastia lamelar anterior profunda com formação bem-sucedida de bolha e dissecção lamelar manual, frente à falha de formação da grande bolha.

**Métodos:**

Este estudo comparativo retrospectivo incluiu 60 olhos de 60 pacientes com ceratocone submetidos à cirurgia de ceratoplastia lamelar anterior profunda. Os pacientes foram agrupados como grande bolha (+) e grande bolha (-) de acordo com o sucesso da formação da grande bolha durante a cirurgia. O grupo grande bolha (+) incluiu 42 olhos, enquanto o grupo grande bolha (-) tinha 18 olhos. Além disso, para a avaliação dos efeitos da disparidade entre alterações individuais nas propriedades biomecânicas da córnea, reagrupamos os pacientes em 0,25 mm e 0,50 mm. Parâmetros biomecânicos da córnea, caracterizados por histerese corneana e fator de resistência corneana foram medidos com o ORA 12 meses após a cirurgia.

**Resultados:**

Não houve diferença estatisticamente significativa entre os grupos grande bolha (+) e grande bolha (-) em relação aos parâmetros biomecânicos da córnea (histerese corneana: 10,06, 10,25, p=0,716/fator de resistência da córnea: 10,15, 10,07, p=0,805, respectivamente). Além disso, os resultados de paquimetria não diferiram estatisticamente entre os dois grupos. A análise de regressão multivariada demonstrou que a histerese da córnea e o fator de resistência corneana estavam associados positivamente com a espessura corneana central (p<0,001/r^2^=0,506, p<0,001/r^2^=0,561 respectivamente). No entanto, o estudo não revelou associação entre qualquer um dos tamanhos de punção e histerese corneana, bem como entre os tamanhos de punção e o fator de resistência corneano (p=0,673, p=0,643).

**Conclusões:**

A histerese da córnea e os valores do fator de resistência da córnea foram comparáveis com formação de grande bolha e dissecção manual lamelar na ceratoplastia lamelar anterior profunda. Assim, a dissecção manual lamelar não foi uma desvantagem, considerando os fatores biomecânicos da córnea.

## INTRODUCTION

Keratoconus (KC) is a bilateral, non-inflammatory, and progressive ectatic disease of the cornea. Various approaches are used to treat KC. Treatment begins with spectacle correction of astigmatism, followed by use of rigid, gas-permeable contact lenses, collagen cross-linking, and intrastromal corneal ring segments. If these treatments do not provide acceptable vision, keratoplasty has been recommended^(1)^. Due to complications associated with penetrating keratoplasty (PK), deep anterior lamellar keratoplasty (DALK) has become the gold standard treatment for KC. DALK surgery has the advantage of preserving the host endothelium and reducing the risk of endothelial graft rejection^(2)^.

The primary aim of KC treatment is to improve eye optics. However, corneal hysteresis, a biomechanical property of the cornea, has recently been investigated in response to the various treatments in patients with KC^(3)^. The development of the Ocular Response Analyzer (ORA; Reichert Ophthalmic Instruments, Inc., Buffalo, NY, USA) allows assessment of the cornea’s biomechanical properties^(4)^. Corneal hysteresis (CH) is shown to be lower in KC^(5,6)^. In addition, when evaluating the effects of PK and DALK, CH is lower in patients with PK compared to those undergoing DALK^(7,8)^.

The present study’s aim was to compare the cornea’s biomechanical properties in patients undergoing DALK surgery with successful big bubble (BB) formation and manual lamellar dissection upon BB failure.

## METHODS

This retrospective comparative study evaluated 60 eyes of 60 KC patients who previously underwent DALK surgery. Participants were recruited from the cornea ser vice of Haydarpasa Numune Research and Education Hospital. Clinical data were extracted from computerized databases of KC patients who underwent DALK surgery between January 2013 and January 2015. These patients were classified as BB (+) and BB (-) according to BB formation during the surgery. The BB (+) group included 42 eyes, and the BB (-) group had 18 eyes. Moreover, to evaluate the effects of disparity between donor and trephine punches on the cornea’s biomechanical properties, we regrouped the patients as 0.50 mm disparity (36 patients [21 BB (+), 15 BB (-)]) and 0.25 mm disparity (24 patients [21 BB (+), 3 BB (-)]).

The presence of advanced KC was determined in all patients via history, slit lamp examination, keratometry, and corneal topography. For all patients, the decision to perform surgery was based on poor visual function and resistance to other optimal optical correction methods. The study excluded patients with hydrops or deep stromal scar, ocular disorders except KC, previous ocular surgery, and systemic disorders. Furthermore, surgeries that were converted to PK and patients with perforation of Descemet membrane (DM) were also excluded from the study. The study was approved by the Clinical Research Ethics Committee of Haydarpasa Numune Education and Research Hospital. The study was performed in accordance with the principles of the Declaration of Helsinki, and all patients provided informed consent.

The cornea’s biomechanical properties, characterized by CH and corneal resistance factor (CRF), were measured using the ORA at the 12-month follow-up. All sutures were removed at least three months before the ORA was performed. ORA measurements were taken while the patients were seated and focusing on the fixation light. All patients received three measurements. The device alignment system allowed positioning the air tube at a precise spot near the corneal apex. The rapid air impulse caused the cornea to move inward when the alignment was completed. Within milliseconds, the air impulse stopped, and the cornea began to move outward to its normal position. Corneal resistance to this air impulse caused delays in the inward and outward movement of the cornea, resulting in two pressure values. CH, which provides information about the viscoelastic structure of the cornea, was calculated by the difference between the two pressure values. CRF, which is the cumulative effect of both the viscous and elastic resistance of the air puff while deforming the cornea, was calculated as a linear function of the two pressures associated with the two applanations. The mean of the three measurements was used for statistical analysis.

Under general anesthesia, DALK was performed using the BB technique described by Anwar and Teichmann^(9)^. A Hessburg-Barron (JedMed Instrument Co., St. Louis, MO, USA) suction trephine (7.00 or 7.5 mm) was used for partial thickness trephination of the host cornea up to a depth of 60%-80%. After the remnants of the posterior stromal lamellae were removed, resulting in a fully transparent DM, attention was focused on preparing the donor tissue. If the BB could not be formed, the procedure was repeated. After several additional failed attempts at formation, a layer-by-layer manual stromal dissection was performed with a blunt tipped spatula to expose a uniform plane as close to the DM as possible. The donor cornea was cut from the underlying endothelium with a 0.25 or 0.50 mm larger (with respect to the recipient cornea) Barron Donor Cornea Punch (Katena Products, Inc., Denville, NJ, USA), and the endothelium was scraped completely from the button. The donor cornea was placed on the recipient bed and sutured into position using four 10-0 monofilament nylon sutures for fixation. Next, the graft was fixed with 16-bite single running sutures.

### Statistical analysis

IBM SPSS Statistics, Version 19.0 (IBM Corp., Armonk, NY, USA) was used for statistical analysis. Continuous variables are presented as mean, median, standard deviation, minimum, and maximum values, and qualitative variables are shown as frequencies and percentages. Normality was assessed using the Shapiro-Wilk test. The Mann-Whitney U test was used to perform comparisons between the two groups for age, pachymetry, follow-up time, suction trephine and donor punch diameters, CRF, and CH. The Pearson chi-square test was used to compare sex differences between the two groups. Multivariate regression analysis was used to evaluate the effects of suction trephine and donor punch diameters and pachymetry on dependent variables of CRF and CH. A p-value <0.05 was considered statistically significant for all statistical comparisons.

## RESULTS

No statistically significant difference between the BB (+) and BB (-) groups was noted in terms of age or sex. In addition, no striking difference was found between the two groups in terms of trephine punch diameters and follow-up periods. However, the donor punch diameter was higher in the BB (+) group, which was statistically significant (p=0.019). All of these findings are shown in table 1.

**Table 1:** Patient characteristics

	Total (n=40)	BB (+) group (n=28)	BB (-) group (n=12)	p-value
Age (years) (mean ± SD)	25.08 ± 5.29	26.98 ± 5.55	23.1 ± 3.70	0.084
Sex				
Male (n)	34	20	14	1.0
Female (n)	28	18	10	
Trephine diameter (mean) (mm)	7.22	7.26	7.12	0.163
Donor punch diameter (mean) (mm)	7.56	7.63	7.41	0.019

BB= Big bubble.

No key difference was seen between the BB (+) and BB (-) groups regarding postoperative the cornea’s biomechanical parameters (CH: 10.06 ± 0.94 mmHg, 10.25 ± 1.80 mmHg; p=0.716/CRF: 10.15 ± 1.66 mmHg, 10.07 ± 2.30 mmHg, p=0.805, respectively). Moreover, postoperative pachymetry results were not distinctly different between the two groups. Postoperatively, the mean central corneal thickness in the BB (+) group was 562.85 ± 10.56 µm, whereas in the BB (-) group, it was 565.33 ± 10.76 µm. Such between-group differences were not statistically significant (p=0.493) (Table 2). In addition, there was no clear difference between the postoperative endothelial cell counts of the BB (+) and BB (-) groups (2739.6 ± 97.5 cells/mm^2^, 2680.7 ± 171.3 cells/mm^2^, respectively; p=0.271). Overall, 36 type 1 BB, 4 type 2 BBs, and 1 type 3 BB were formed. Statistical analysis could not be performed due to the low number of type 2 and 3 BBs.

**Table 2:** Corneal biomechanical values in the BB (+) and BB (-) groups at 12 months after DALK surgery

	BB (+) (n=42) (mean ± SD)		BB (-) (n=18) (mean ± SD)		p-value
CH, mmHg	10.06 ± 0.94		10.25 ± 1.80		0.716
CRF, mmHg	10.15 ± 1.66		10.07 ± 2.30		0.805
Pachymetry, µm	562.85 ± 10.56		565.33 ± 10.76		0.493

BB= Big bubble; CH= Corneal hysteresis; CRF= Corneal resistance factor; DALK= Deep anterior lamellar keratoplasty.

Multivariate regression analysis revealed a positive association between CH and CRF values and postoperative central corneal thickness (CCT) (p<0.001/R^2^=0.506; p<0.001/R^2^=0.561, respectively). However, the study did not demonstrate an association between vacuum trephine size and CH or vacuum trephine size and CRF (p=0.673, p=0.643). Moreover, we evaluated the effect of donor punch size on the CH results and found no association between donor punch size and CH or donor punch size and CRF (p=0.548, p=0.947).

We also investigated the effect of disparity between trephine and donor punch sizes on biomechanical properties. We regrouped the patients into 0.5 mm and 0.25 mm disparity groups according to the trephine and donor punch sizes. The CH and CRF values in the 0.5 mm disparity group were 10.22 ± 1.31 mmHg and 10.10 ± 1.96 mmHg, respectively. Likewise, CH and CRF values in the 0.25 mm disparity group were 9.99 ± 1.20 mmHg and 10.18 ± 1.85 mmHg, respectively. There was no statistically significant difference in mean CH and CRF between the two groups (p=0.895).

## DISCUSSION

The ORA device allows objective measurement of the cornea’s biomechanical properties, such as elasticity and viscosity. Many studies have revealed decreased CH in KC patients^(5,6,10)^. After presentation of the findings of CH in KC patients, the results of treatment modalities in KC also attracted attention. In the present study, we attempted to evaluate the effect of lamellar dissection due to unsuccessful BB formation on the cornea’s postoperative biomechanical properties, revealing no significant difference between the BB (+) and BB (-) groups. In addition, CH and CRF results were positively associated with CCT, but not with vacuum trephine or donor punch size. Based on these results, it appears that DALK surgery with unsuccessful BB formation had no postoperative disadvantages in terms of CH.

By evaluating the changes in KC patients undergoing PK, Yenerel et al. demonstrated a beneficial effect of PK on the cornea’s biomechanical properties. They found that CH and CRF values almost reached the values of normal eyes in patients with PK. Moreover, they stated that CH and CRF values decreased in KC patients, including in those with forme frust and manifest keratoconus, when compared to normal subjects (CH values: 9.21 ± 1.38 mmHg, 8.19 ± 1.49 mmHg, and 11.43 ± 1.52 mmHg; CRF values: 8.21 ± 1.64 mmHg, 6.79 ± 1.81 mmHg, and 9.94 ± 2.34 mmHg, respectively)^(11)^. A study by Feizi et al. analyzed the effect of anatomical features on the transplanted corneas’ biomechanical properties, revealing that both CH and CRF values were associated with donor size and corneal graft thickness. Patients with large donor tissues had higher biomechanical values (CH: p=0.025, R^2^=0.35/CRF: p=0.001, R^2^=0.51)^(3)^. In this study, we also found a correlation between graft pachymetry results and CH and graft pachymetry results and CRF (p<0.001/R^2^=0.506; p<0.001/R^2^=0.561, respectively). However, our study demonstrated no correlation between the cornea’s biomechanical properties and vacuum trephine or the biomechanical properties and graft size (p=0.673, p=0.643, p=0.548, and p=0.947, respectively). This difference may be due to the use of different surgical procedures.

In a recent study, we compared the biomechanical properties of the cornea in KC patients who had either PK or DALK surgery together with the control subjects. We found that DALK surgery resulted in higher biomechanical values in the cornea compared to PK surgery. CH values were 10.57 ± 1.40 mmHg in the control group, 10.15 ± 1.23 mmHg in the DALK group, and 8.29 ± 1.41 mmHg in the PK group. We also determined that DALK surgery offered better corneal biomechanical results^(8)^. Hosny et al. also investigated the changes in corneal biomechanics after performing different keratoplasty techniques, and found that patients with PK had lower biomechanical properties compared to those undergoing DALK. CH values in the control, PK, and DALK groups were 10.86 ± 1.36 mmHg, 9.57 ± 0.33 mmHg, and 10.87 ± 1.39 mmHg, respectively^(7)^. In a recent study, Maeda et al. investigated the cornea’s biomechanical properties in three corneal transplantation techniques using a new device called the dynamic Scheimpflug analyzer. The highest deformation amplitude (DA) was found in the PK group (DA: 1.20 ± 0.13 mm). In DALK patients, the DA value was higher in the control group (DA: 1.07 ± 0.09 mm) and lower than in the PK group (1.18 ± 0.18 mm). The results using a new device were also compatible with those of former studies^(12)^. It was hypothesized that preserved host DM and endothelium were the reasons for the improved corneal biomechanical values. Clearly, treating KC patients with any keratoplasty technique also treats the patient’s biomechanical properties. In our study, manual lamellar dissection due to unsuccessful BB formation did not result in worse corneal biomechanical properties.

In the present study, we also compared the postoperative biomechanical properties of the corneas in KC patients who had DALK surgery to successful BB formation or lamellar dissection due to unsuccessful BB for mation. Host DM and endothelium were preserved in both surgeries. In this study, no statistically significant difference was found between the BB (+) and BB (-) groups. Thus, lamellar dissection was not a disadvantage in terms of corneal biomechanical factors. Moreover, the size of the graft did not affect the cornea’s biomechanical properties.

The present study is the first to assess biomechanical values in subgroups of patients undergoing DALK. However, some study limitations should be addressed. The major limitation was the lack of stromal thickness values and a comparison of hysteresis according to residual stromal thickness. Preoperative CH and CRF outcomes were not measured in all of the patients; hence, we could not compare the preoperative with the postoperative results. The retrospective nature of the study was another obstacle. Nonetheless, our study revealed no difference in the cornea’s biomechanical properties between successful BB formation and lamellar dissection due to unsuccessful BB formation in DALK surgery.
